# Long Non-Coding RNA-ROR Mediates the Reprogramming in Cardiac Hypertrophy

**DOI:** 10.1371/journal.pone.0152767

**Published:** 2016-04-15

**Authors:** Feng Jiang, Xiangyu Zhou, Jing Huang

**Affiliations:** 1 Department of Cardiology, the Second Affiliated Hospital of Chongqing Medical University, Chongqing, China; 2 Department of Vascular and Thyroid Surgery, the Affiliated Hospital of Southwest Medical University, Luzhou, Sichuan, China; University of Cincinnati, College of Medicine, UNITED STATES

## Abstract

**Background:**

Cardiac hypertrophy associated with various cardiovascular diseases results in heart failure and sudden death. A clear understanding of the mechanisms of hypertrophy will benefit the development of novel therapies. Long non-coding RNAs (lncRNAs) have been shown to play essential roles in many biological process, however, whether lncRNA-ROR plays functional roles in the reprogramming of cardiomyocyte remains unclear.

**Methodology/Principal Findings:**

Here we show that lncRNA-ROR plays important roles in the pathogenesis of cardiac hypertrophy. In hypertrophic heart and cardiomyocytes, the expression of lncRNA-ROR is dramatically increased, downregulation of which attenuates the hypertrophic responses. Furthermore, the expression of lncRNA-ROR negatively correlates with miR-133, whose expression is increased when lncRNA-ROR is knocked down. In line with this, overexpression of miR-133 prevents the elevation of lncRNA-ROR and re-expression of ANP and BNP in cardiomyocytes subject to phenylephrine treatment.

**Conclusions/Significance:**

Taken together, our study demonstrates that lncRNA-ROR promotes cardiac hypertrophy via interacting with miR-133, indicating that lncRNA-ROR could be targeted for developing novel antihypertrophic therapeutics.

## Introduction

Cardiac hypertrophy, characterized by the increased mass of the heart, is the compensatory remodeling of the heart in response to mechanical stress or neurohormonal stimulation [1]. This phenomenon is present in a variety of cardiovascular diseases, including hypertension, valvular disease, and heart failure. The prolonged ventricular hypertrophy is associated with increased risk of developing heart failure and malignant arrhythmia leading to high morbidity and mortality [2]. Understanding of the mechanisms underlying the hypertrophy will help to develop novel and effective therapeutics for the reverse of this process and increase the quality of life of patients.

Cardiomyocyte is the dominating cell type in the process of cardiac remodeling. At the cellular level, the increased protein synthesis and heightened organization of the sarcomere are hallmarks of cardiomyocyte hypertrophy. Gene expression reprogramming is the base of pathological cardiomyocyte hypertrophy. During the pathological hypertrophy, the re-expression of fetal genes contributes to the molecular and cellular events involved in the growth of myocytes, such as atrial natriuretic peptide (ANP), brain natriuretic peptide (BNP), and skeletal α-actin [3,4,5]. Accumulating studies have suggested the significant roles of many transcriptional factors, such as the GATA family transcriptional factors, myocyte enhancer factor 2 (MEF2) transcriptional factors, and the homeobox transcriptional factor, in the re-expression of these fetal genes and cardiomyocyte hypertrophy [6].

In addition to transcriptional factors, RNAs that do not code for proteins, namely non-coding RNAs, have been widely investigated in the regulation of gene expression. Non-coding RNAs are divided into two major groups based on size: the long non-coding RNAs (lncRNAs) with more than 200 bases in length, and small non-coding RNAs, which have less than 200 nucleotides including microRNAs and short interfering RNAs [7]. Both lncRNAs and microRNAs have been shown to play essential roles in the development of cardiac hypertrophy [8,9]. Unlike cardiac microRNAs, which received extensive studying, the role of cardiac lncRNAs remains largely unrevealed especially in cardiac hypertrophy despite the presence of more than 300 lncRNAs in the myocardium [10]. LncRNA ROR was firstly was identified as a human large intergenic non-coding RNA ROR (LINC-ROR), which is located at 18q21.31 in chromatin, and also called lincRNA-ST8SIA3, or ROR. Recently, human lncRNA-ROR has been shown to modulate reprogramming and turn lineage-committed cells into induced pluripotent stem cells [11]. Several studies also revealed functions of LINC ROR in regulating Oct4, Nanog, and Sox2 in human embryonic stem cell self-renewal as a miRNA sponge[12], and suppressing p53 in response to DNA damage[13]. However, whether lncRNA-ROR plays functional roles in the reprogramming of cardiomyocyte and ultimately cardiac hypertrophy remains unclear.

In this study, we demonstrated that the expression of lncRNA-ROR was increased in hypertrophic heart and cardiomyocytes. Knockdown of lncRNA-ROR reversed the hypertrophic responses in cardiomyocytes. Furthermore, the pro-hypertrophic effect of lncRNA-ROR was mediated via decreasing the expression of another non-coding RNA miR-133, overexpression of which attenuated lncRNA-ROR expression and fetal gene expression. Our study reveals a novel mechanism underlying cardiac hypertrophy, which could be targeted for the development of antihypertrophic therapeutics.

## Materials and Methods

### Mouse model of cardiac hypertrophy

Male C57BL6 mice (20–25 g) were purchased from Vitalriver Laboratory Animal Company (Beijing, China). All animals were housed under pathogen-free conditions and kept on standard mouse chow with free access to tap water. This study conformed to the Guide for the Care and Use of Laboratory Animals published by the US National Institutes of Health. Furthermore, the present work plan was also approved by the research ethics committee of Chongqing Medical University. Transverse aortic constriction (TAC) was carried out to promote pressure overload-induced cardiac hypertrophy in mice. Briefly, age matched mice were anesthetized with ketamine (100 mg/kg) and xylazine (10 mg/kg), placed on a ventilator (Harvard Rodent Ventilator, Harvard Apparatus, Holliston, MA) and core temperature was maintained at 37°C with a heating pad. Stenosis of the transverse aorta was measured using anatomic M-mode echocardiography from mice with satisfactory echocardiographic images of the aortic arch. Sham-operated mice underwent the same operation, without suture tied around the aorta.

### Cardiomyocyte culture

Primary culture of neonatal cardiomyocytes were prepared as described [14]. Briefly, hearts from 1 to 3-day-old Sprague–Dawley rats were dissected and the ventricles were minced into small pieces. The tissue was transferred to digestion solution containing 0.1% collagenase type IV, 0.1% trypsin, 15 μg/ml DNase I, and 1% chicken serum in HEPES-buffered saline and incubated at 37°C for 5–6 15-min periods. After the digestion, 10% calf serum was added to neutralize trypsin. After centrifugation, the dissociated cells were resuspended in Dulbecco’s modified Eagle’s medium/F12 with supplements and plated onto the collagen-coated silicone sheet. The culture medium was replaced with a serum-free medium 24–36 hrs after seeding and incubated for at least another day before used. Transfection of primary myocyte culture was carried out with Lipofectamine 2000 according to manufacturer’s instructions.

### Transfection of miRNA precursors and inhibitors

The miR-133 Mimics (Ribo Company) are chemically modified, single-stranded nucleic acids designed to specifically enhance the expression of miR-133. The nucleic acids with scramble sequence was used as the negative control (NC). Depending on the experiment, NC and Mimics at a final concentration of 50 nM or 100 nM were transfected with Lipofectamine 2000 (Invitrogen) into cardiomyoctyes or HeLa cells cultured in 24-well plates (BD Biosciences) according to the oligonucleotide manufacturer’s protocol.

### Construct of luciferase reporter system and transfection in Hela cells

The luciferase reporter plasmid pMIR-REPORT-luciferase was applied to clone the reporter gene. One luciferase reporter plasmid covering the 3’UTR of lncRNA-ROR sequence was constructed by Ribo Company (Guangzhou, Guangdong, China). The mutation of GGGGACC to GGGGAAA was constructed by Transgene Company (Beijing, China). Luciferase reporter plasmids were transfected into the cells with Renilla luciferase plasmid. Briefly, HeLa cells were cotransfected in 12-well plates with 50 nM or 100 nM NC or Mimics and 0.4 μg of the 3’UTR luciferase reporter vector. Cell lysates were prepared 48 hours later; luciferase activity was measured with a Monolight 3010 luminometer (Pharmingen, San Diego, Calif) and expressed as relative light units with a luciferase assay kit (Promega, Madison, Wis). All transfections were performed in triplicate from three independent experiments.

### Quantitative reverse transcription-polymerase chain reaction (qRT-PCR)

The total RNA was extracted using the Trizol reagent (Invitrogen, Carlsbad, CA). 1 μg of total RNA was treated with DNase I (Invitrogen) and the cDNA was synthesized in vitro from the mRNA template using SuperScript® III First-Strand Synthesis Kit (Invitrogen). The qPCR was performed using specific primer pairs. The sequences of primers are as follows (from 5’ to 3’): ANP, CAACGCAGACCTGATGGATTT, AGCCCCCGCTTCTTCATTC; BNP, TGGAAACGTCCGGGTTACAG, CTGATCCGGTCCATCTTCCT; lncRNA-ROR, CCTGTCCTCCTGCTCTTTGC, TTTCTTGGGCTGGTTGGTCT; GAPDH, GGAGCGAGATCCCTCCAAAAT, GGCTGTTGTCATACTTCTCATGG. All of the microRNAs primers for reverse transcription or amplification were purchased from Ribo Biotech (Guangzhou, Guangdong, China) including miR-21, miR-208, miR-499, miR-1, miR133, miR-30 and U6. The fold change of each gene was calculated using 2^-ΔCt^, where ΔCt = Ct_target_-Ct_GAPDH_ for ANP, BNP, lncRNA-ROR; or ΔCt = Ct_target_-Ct_U6_ for miR-21, miR-208, miR-499, miR-1, miR133, and miR-30.

### Western blot

Cultured cells or heart tissue were lysed on ice by mammalian protein extraction reagent (ThermoFisher Scientific, Waltham, MA, USA) plus protease inhibitors (Sigma-Aldrich, St. Louis, MO, USA). After centrifugation, protein (40 μg) for each cell lysate was separated by SDS-PAGE and transferred onto PVDF membranes (Bio-Rad). Membranes were blocked with 5% dry milk in TBST and immunoblotted with primary antibodies as follows: ANP (mouse monoclonal, dilution of 1:1000, Santa Cruz) and BNP (mouse monoclonal, dilution of 1:1000, Santa Cruz). GAPDH serves as loading control in every experiments. HRP conjugated secondary antibodies (Jackson ImmunoResearch Laboratories, West Grove, PA, USA) and enhanced chemiluminescence (Pierce, Rockford, IL, USA) were used to detect the protein bands. Digital images of luminescence were taken by AlphaImager (FluorChem5500TM, Alpha Innotech, San Leandro, CA).

### Immunofluorescent staining

Cultured cells were rinsed with PBS twice and fixed with 4% paraformaldehyde for 20 min and washed with PBS three times. Then, the fixed cells were blocked with 10% normal goat serum plus 1% BSA (Sigma-Aldrich) for 45 min, and incubated with anti-α-actinin (mouse monoclonal, dilution of 1:1000, Sigma-Aldrich) at 4°C in the dark overnight. After washing three times with PBS, cells were further incubated with TRITC-modified secondary antibody for 1 h at 37°C. Subsequently, the slides were cover slipped with mounting medium (Dako) containing DAPI to counter stain the nuclei.

### Statistics

Data are presented as mean ± s.e.m. of at least three independent experiments. Significance of means between two groups is determined by student’s t-test. A P-value of < 0.05 was considered significantly different.

## Results

### Enhanced expression of lncRNA-ROR in hypertrophic cardiomyopathy

**Fig 1:**
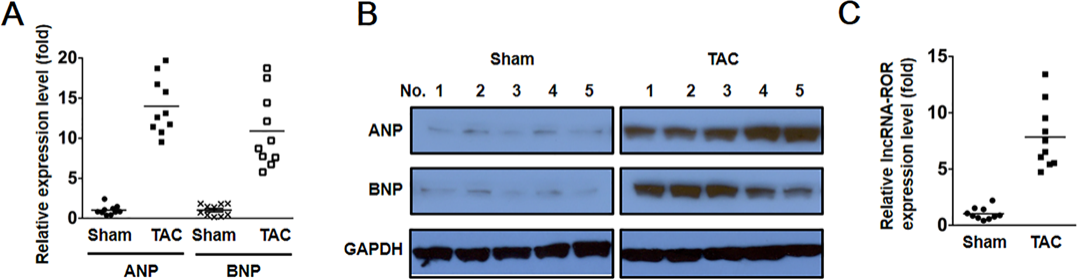
Increased lncRNA-ROR in hypertrophic heart in vivo. (A) Relative mRNA expression levels of ANP and BNP in sham group (Sham) and TAC group (TAC) determined by quantitative RT-PCR. (B) Western blot showing the protein expression of ANP and BNP in control and hypertrophic heart. GAPDH serves as loading control. (C) Relative mRNA expression of lncRNA-ROR in the mouse hearts of Sham group and TAC group.

To investigate the role of lncRNA-ROR in hypertrophic cardiomyopathy, we examine expression of lncRNA-ROR in a mouse model of cardiac hypertrophy. Consistent with previous studies [15], after the induction, the expression of fetal genes ANP and BNP were significantly increased in the heart of TAC group mice compared with that in Sham group as evidenced by RT-PCR and western blot (Fig 1A and 1B). More importantly, the expression level of lncRNA-ROR was also found to be dramatically increased in the heart of mice subject to TAC treatment (Fig 1C).

### Increased expression of lncRNA-ROR in cultured cardiomyocytes treated with PE

**Fig 2:**
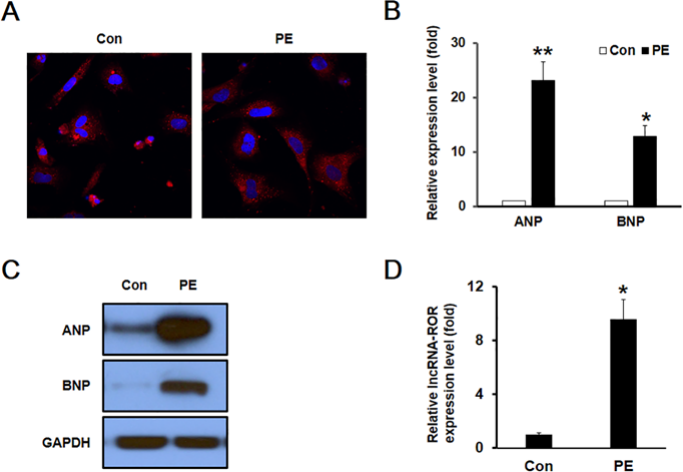
Increased lncRNA-ROR in hypertrophic cardiomyocytes cultured in vitro. (A) Immunocytochemical staining with anti-α-actinin shows the enlargement of cultured cardiomyocytes after PE treatment. The mRNA (B) and protein (C) expression levels of ANP and BNP were increased in PE-induced hypertrophic cardiomyocytes as determined by quantitative RT-PCR and western blot, respectively. (D) Relative mRNA expression of lncRNA-ROR in the cultured cardiomyocytes after PE treatment. * p<0.05, ** p<0.01 as compared with the control.

To explore the role of lncRNA-ROR in vitro, we examined the expression of lncRNA-ROR in an in vitro model of cardiomyocyte hypertrophy generated by treating cultured neonatal cardiomyocytes with 20 μM phenylephrine (PE), which is widely employed to induce cardiomyocyte hypertrophy [16,17]. As shown in Fig 2A, the size of cultured cardiomyocytes was dramatically increased following the stimulation with PE. Similar to the in vivo study, the expression of fetal genes ANP and BNP was also significantly increased in PE-treated cardiomyocytes at mRNA and protein level (Fig 2B and 2C). These data indicated that PE induced hypertrophy of cardiomyocytes in vitro. RT-PCR analysis revealed that the expression of lncRNA-ROR was also dramatically increased in the in vitro model of cardiomyocyte hypertrophy (Fig 2D).

### Suppression of lncRNA-ROR attenuated hypertrophic changes of cardiomyocytes

**Fig 3:**
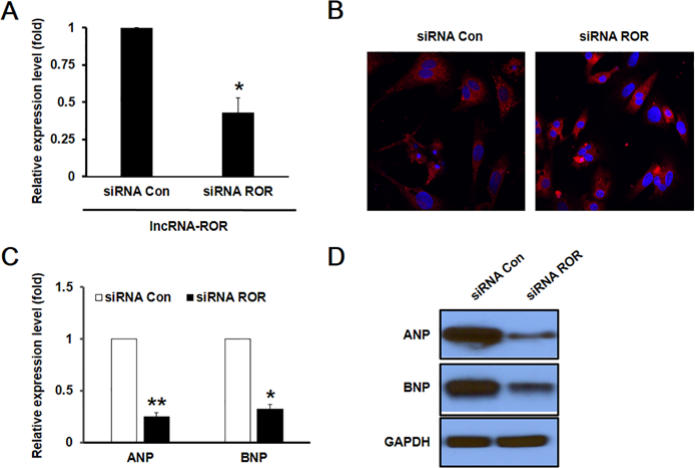
Knockdown of lncRNA-ROR reversed hypertrophic responses of cardiomyocytes. (A) After the transfection of ROR-specific siRNA, the mRNA expression of lncRNA-ROR was dramatically decreased compared with the control siRNA-transfected cells. (B) The enlarged cardiomyocytes induced by PE treatment was attenuated by the transfection of lncRNA-ROR. The mRNA (C) and protein (D) expression levels of ANP and BNP were decreased after the knockdown of lncRNA-ROR. * p<0.05, ** p<0.01 compared with control siRNA-treated cells.

To further investigate the role of lncRNA-ROR in cardiomyocyte hypertrophy in vitro, we knocked down the expression lncRNA-ROR with ROR specific siRNA prior to stimulation with PE. Transfection with ROR siRNA resulted in a significantly decreased expression of lncRNA-ROR (Fig 3A). Concurrently, PE-induced increase in cell size was markedly attenuated by ROR siRNA transfection (Fig 3B), indicating that the synthesis of protein was slowed. In addition, the PE-induced accelerated expression of embryonic ANP and BNP was also dramatically attenuated compared with control siRNA transfected cardiomyocytes (Fig 3C and 3D).

### MicroRNA-133 negatively correlated with lncRNA-ROR in PE-induced cardiomyocyte hypertrophy

**Fig 4:**
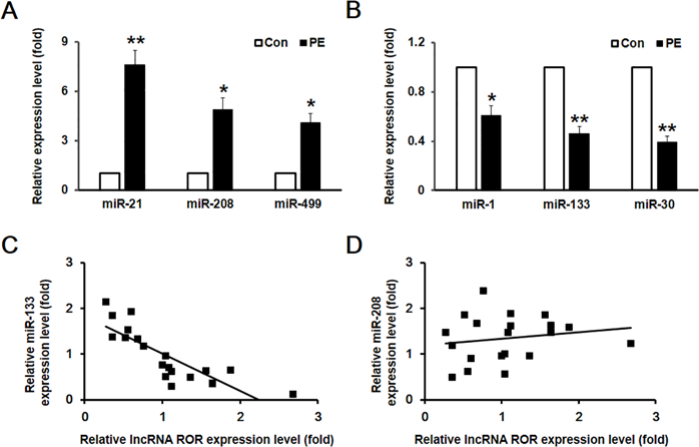
MicroRNA 133 negatively correlated with lncRNA-ROR. (A) Relative mRNA expression levels of miR-21, miR-208, and miR-449 were increased in cultured cardiomyocytes after PE treatment. (B) Relative mRNA expression levels of miR-1, miR-133, and miR-30 were decreased in cultured cardiomyocytes after PE treatment. Plotting the expression of miR-133 (C) or miR-208 (D) against that of lncRNA-ROR in the heart of mice model of cardiac hypertrophy showed that there was no correlation between miR-208 and lncRNA-ROR, however, miR-133 negatively correlated with lncRNA-ROR. * p<0.05, ** p<0.01 compared between control and PE-treated cells.

Altered expression of miRNAs has been shown to contribute to the pathogenesis of many cardiovascular disease conditions including cardiac hypertrophy [9,18]. In this study, we examined the expression of specific ventricular miRNAs in PE-induced ventricular myocyte hypertrophy, including miR-1, miR-133, miR-208, miR-499. Our data showed that hypertrophic cardiomyocytes exhibited increased expression of miR-208 and miR-499 and decreased expression of miR-1, miR-133, in which miR-21 and miR-30 were used as the positive control (Fig 4A and 4B). Plotting the expression levels of miR-208 or miR-133 over that of lncRNA-ROR revealed that a negative correlation existed between miR-133 and lncRNA-ROR, but no correlation between miR-208 and lncRNA-ROR (Fig 4C and 4D). These results suggest that lncRNA-ROR may exert the pro-hypertrophic effect via interacting with miR-133.

### Long non-coding RNA-ROR served as miR-133 sponge

**Fig 5:**
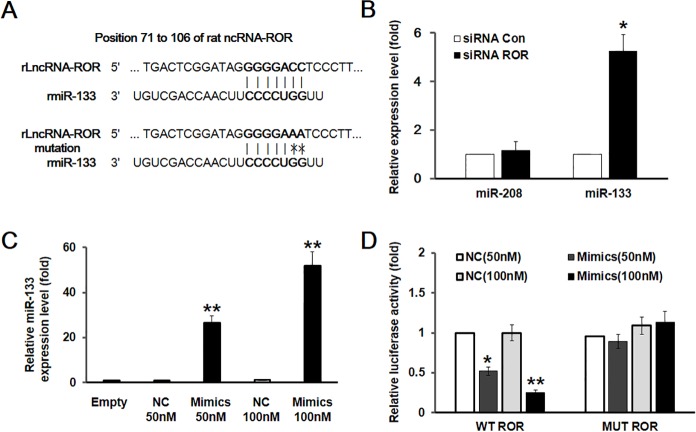
lncRNA-ROR functionally interacted with miR-133. (A) Sequence alignment analysis revealed the interaction site of miR-133 in rat lncRNA-ROR. In the mutated lncRNA-ROR, the interaction site was mutated. (B) The expression of miR-133, but not miR-208 was significantly increased after the transfection with ROR-specific siRNA. (C) RT-PCR showed that the expression of miR-133 was increased when it was overexpressed in Hela cells. NC represents negative control. (D) Luciferase activity in cells transfected with luciferase reporter plasmid containing wild type or mutated ROR with or without overexpression of miR-133. * p<0.05, ** p<0.01.

It is well known that miR-133 play important roles in cardiac hypertrophy. However, the targets of miR-133 and the functional interaction between them have received increasing attention for understanding the role of miR-133 [19]. The negative correlation between the expression of lncRNA-ROR and miR-133 suggest that an interaction may exist between them. Indeed, the sequence alignment located the binding site for miR-133 in rat lncRNA-ROR (Fig 5A). Furthermore, knocking down lncRNA-ROR dramatically increased the expression of miR-133, but not miR-208 (Fig 5B). To examine the effect of miR-133 on lncRNA-ROR-mediated function, miR-133 was successfully overexpressed with transfecting miR-133 Mimics to cardiomyocytes (Fig 5C). Next, we applied luciferase reporter system in which wild type or mutated lncRNA-ROR was inserted. As shown in Fig 5D, overexpression of miR-133 dramatically decreased wild type lncRNA-ROR, but not the mutated lncRNA-ROR-induced luciferase activity. These results suggest that lncRNA-ROR functionally interacts with miR-133.

### MicroRNA-133 reversed the pro-hypertrophic effect of lncRNA-ROR

**Fig 6:**
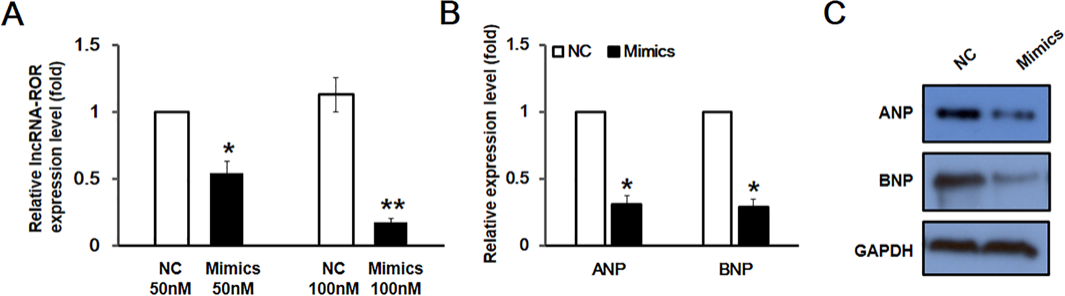
Overexpression of miR-133 attenuated hypertrophic response. (A) RT-PCR showed that the expression of lncRNA-ROR was decreased by overexpression of miR-133 in a concentration-dependent manner. Relative expression levels of mRNA (B) or protein (C) of ANP and BNP were decreased by overexpression of miR-133. NC represents negative control. * p<0.05, ** p<0.01.

To investigate whether the pro-hypertrophic effect of lncRNA-ROR could be manipulated by targeting miR-133, we overexpressed miR-133 in hypertrophic cardiomyocytes. As shown in Fig 6A, overexpression of miR-133 successfully attenuated the expression of lncRNA-ROR in cardiomyocyte hypertrophy. Consistently, the expression of fetal gene ANP and BNP was also markedly decreased by overexpression of miR-133 as evidenced by RT-PCR and western blot (Fig 6B and 6C), indicating that the pro-hypertrophic effect of lncRNA-ROR can be attenuated by targeting miR-133.

## Discussion

Cardiac hypertrophy involves the growth of myocytes as a result of re-expression of fetal genes and enhanced protein synthesis. Long non-coding RNAs and microRNAs are critical for regulating the gene expression and have been shown to play important roles in the pathogenesis of cardiovascular diseases including cardiac hypertrophy [8]. A recent study reveals that the lncRNA *Mhrt* protects the heart from pathological hypertrophy via interfering with the binding of a chromatin remodeling factor Brg1 to chromatinized DNA targets [20]. In addition to the protective role of lncRNA, we demonstrate in the present study that the newly identified lncRNA-ROR promotes cardiac hypertrophy by showing that the expression of lncRNA-ROR is dramatically increased in both hypertrophic heart and in vitro cultured cardiomyocytes subject to PE treatment, which are associated with increased expression of fetal genes ANP and BNP. Increased expression of lncRNA-ROR may reflect the compensatory response to hypertrophy instead of the cause of hypertrophy. However, this may not be the case, as suppression of lncRNA-ROR reversed the hypertrophic responses of cardiomyocytes in response to PE. These results clearly demonstrate that lncRNA-ROR promotes the re-expression of fetal genes and growth of cardiomyocytes contributing to hypertrophic cardiomyopathy.

In addition to lncRNAs, the roles of miRNAs in the pathogenesis of cardiac hypertrophy have been extensively studied. Numerous miRNAs have been identified to be differentially expressed in hypertrophic hearts. Some of these miRNAs are upregulated and promote the cardiac hypertrophy and heart failure, such as miR-21, miR-208, and miR-499, while others are downregulated in hypertrophic hearts, such as miR-1, miR-133, and miR-30 [21,22]. In line with these studies, we also detected increase of miR-21, miR-208, and miR-499 in the in vitro model of cardiomyocyte hypertrophy as well as the decreased expression of miR-1, miR-133, and miR-30. Previous study demonstrates that lncRNA-ROR promotes the self-renewal of embryonic stem cells via repressing the repressor miR-145 [12]. In the present study, we demonstrate that lncRNA-ROR negatively correlate with the expression of miR-133, but not other miRNAs examined, suggesting that like the interaction between lncRNA-ROR and miR-145, lncRNA-ROR may serve as the sponge of miR-133. Indeed, our data showed that the expression of miR-133 was dramatically increased when knocking down lncRNA-ROR. Furthermore, we identified the interaction site of miR-133 in the sequence of lncRNA-ROR, mutation of which abolished the repression of lncRNA-ROR-mediated luciferase activity induced by over expression of miR-133. These results strongly support the idea that lncRNA-ROR interacts with miR-133.

Decreased expression of miR-133 in cardiac hypertrophy suggests that miR-133 may protect the heart from pathological hypertrophy. Indeed it has been shown that over expression of miR-133 resulted in significant reduction of the size of cardiac myocytes and expression of fetal genes while loss function of miR-133 led to increase of cardiac myocytes and upregulation of cardiac hypertrophy markers [23]. Here we further show that overexpression of miR-133 decreases the expression of lncRNA-ROR in addition to the decrease of fetal genes ANP and BNP. Our present study advanced the understanding of the mechanism underlying the protective effect of miR-133 on hypertrophic cardiomyopathy. We speculate that miR-133 serve as the lncRNA-ROR sponge and attenuates the pro-hypertrophic effect of lncRNA-ROR.

In conclusion, we demonstrate that increased lncRNA-ROR promotes the re-expression of fetal genes and cardiomyocyte hypertrophy via inhibiting the expression and function of miR-133. This study indicates that lncRNA-ROR could be targeted for the development of novel anithypertrophic therapeutics.
